# Histomorphometric and immunohistochemical analysis of infectious agents, T-cell subpopulations and inflammatory adhesion molecules in placentas from HIV-seropositive pregnant women

**DOI:** 10.1186/1746-1596-6-101

**Published:** 2011-10-24

**Authors:** Emanuele Baurakiades, Ana PC Martins, N Victor Moreschi, Camila DA Souza, Karla Abujamra, Augusto O Saito, Maíra C Mecatti, Mônica G Santos, Camilla R Pimentel, Larissa LG Silva, Cristina R Cruz, Lucia de Noronha

**Affiliations:** 1 Center of Health and Biological Sciences, Pontifícia Universidade Católica do Paraná, (Imaculada Conceição), Curitiba, (80215-901), Brazil; 2 Laboratory of Experimental Pathology of Center of Health and Biological Sciences, Pontifícia Universidade Católica do Paraná, (Imaculada Conceição), Curitiba, (80215-901), Brazil; 3 Pathological Anatomy Department, Universidade Federal do Paraná, (General Carneiro), Curitiba, (80060-900), Brazil; 4 Pediatric Department, Institute: Universidade Federal do Paraná, (General Carneiro), Curitiba, (80060-900), Brazil

**Keywords:** HIV, p24, CD4, CD8, ICAM, VCAM, placenta

## Abstract

**Background:**

The aim of this study was to compare histomorphometric changes and the results of immunohistochemical tests for VCAM, ICAM-1, CD4 and CD8 in normal placentas from HIV-seropositive pregnant women.

**Methods:**

Samples of normal placentas were divided into 2 groups: healthy HIV-seronegative pregnant women (control group = C = 60) and HIV-seropositive women (experimental group = E = 57). Conventional histological sections were submitted to morphometric analysis and evaluated in terms of the immunohistochemical expression of ICAM-1, VCAM, CD4 and CD8.

**Results:**

The villi in group E were smaller than those in group C. The median for the CD8+ T cell count was higher in group E than in group C (p = 0.03). Immunohistochemical expression of ICAM-1 was observed in 57% of the cases in group E, compared with 21% of those in group C (p = 0.001). There was no difference in VCAM expression or CD4+ cell counts between groups and no correlation between the data for antiretroviral therapy and morphometric or immunohistochemical data.

**Conclusions:**

The morphometric data showed that placentas of HIV-seropositive pregnant women tend to have smaller villi than those of seronegative women. In addition, immunohistochemical testing for infectious agents helped to identify cases that were positive for microorganisms (6/112) that routine pathological examination had failed to detect. The anti-p24 antibody had a limited ability to detect HIV viral protein in this study (2/57). Correlation of immunohistochemical expression of CD8+ T cells and ICAM-1 with the presence of HIV in the placenta revealed that those expressions can act as biomarkers of inflammatory changes. There was no correlation between the data for antiretroviral therapy and morphometric or immunohistochemical data.

## Introduction

Vertical transmission of HIV is the main route of HIV infection in children. In Brazil this route is responsible for more than 80% of HIV cases in children under 13 years of age and for almost all cases currently diagnosed in this age group. By June 2004, a total of 9162 cases of vertically transmitted HIV had been reported in Brazil, or 2.5% of all AIDS cases reported in this country[1].

Although the placenta acts as a barrier to vertical transmission, trophoblasts can become infected through cell-to-cell transmission during this first phase because they are in close contact with maternal blood vessels[2].

The first contact between trophoblast and decidual cells, and hence with maternal blood, occurs during the first trimester. However, in utero transmission of HIV during the first trimester is rare[2].

Although the placenta provides a natural barrier to HIV-1 infection, the risk of vertical transmission increases in the presence of chorioamnionitis and other chronic infections, such as syphilis, toxoplasmosis, hepatitis B and C and infections caused by viruses from the family Herpesviridae, which are known to be responsible for altering the placental barrier[3,4].

Pathological examination of placentas in HIV-seropositive women can help in the diagnosis of acute and chronic infections. However, these placentas can have minimal alterations or even be histologically normal. Small increases in the number of macrophages and T cells in the villous stroma, thickening of the basement membrane in the syncytiotrophoblast and foci of necrosis in the placental villi have been described[5].

This study aimed to analyze normal placentas from HIV-seropositive women by correlating data on villous morphometry and immunohistochemical findings with data from the medical records of the respective pregnant women and their newborns.

## Methods

Formalin-fixed paraffin-embedded samples of normal placentas from HIV-seropositive women who gave birth between the years 2003 and 2005 at the Hospital de Clínicas, Federal University of Paraná (HC-UFPR) were used.

Four histological sections from all the placentas were reviewed and found to be histologically normal. We did not observe pathological lesions or maturation changes, and weights of the newborns were normal for gestational age. The placentas were from pregnant women who had been diagnosed with HIV before or during their pregnancy, and whose newborns had been followed up until the state of their HIV infection had been defined. Patients who abandoned the follow-up before HIV status had been defined were excluded from the study. Placentas from seropositive women who presented with maternal/fetal diseases, such as arterial hypertension, hypertensive disorders of pregnancy, diabetes mellitus, gestational diabetes, negative Rh factor and intrauterine growth retardation were also excluded. Infants with two viral RNA measurements below the detection threshold were considered to be uninfected. Children with two consecutive viral RNA measurements above the threshold were considered to be infected.

The sample was made up of 116 cases, 57 of which were HIV-seropositive (the study group) and 60 seronegative (the control group, with the same inclusion/exclusion criteria as the study group). The samples in both groups were paired by gestational age, which varied from 25 to 42 weeks.

Serological and therapeutic data for the women and their newborns (age and viral load of the mother, antiretroviral therapy administered to the mother and gestational age, as well as the weight and current serological status of the newborn) were obtained from medical records for subsequent analysis together with morphometric and immunohistochemical data.

All the cases in the study were examined under an optical microscope using hematoxylin eosin-stained slides with samples covering the whole thickness of placenta. The villi were analyzed morphometrically at 20× magnification using an Olympus^® ^BX50 binocular microscope attached to a DELL^® ^computer running Image Pro Plus^® ^software. Morphometry consisted of linear measurements of the perimeter and area of the villous surface based on a freehand drawing. The villi of the central portion of the placenta were used for this purpose; the chorionic plate and marginal villi were not used. The sampling error was calculated to determine the number of villi to be measured for each case (n = 116). A total of 100 villi were measured and an average of the 100 measurements was used.

For the immunohistochemical tests, histological sections were used to detect infectious antigens, lymphocyte subpopulations and inflammatory adhesion molecules by means of immunoperoxidase staining. Monoclonal anti-p24 (Dako^®^) and anti-cytomegalovirus (Zymed^®^) antibodies and polyclonal rabbit anti-*Toxoplasma gondii *(ABR^®^), anti-*Treponema pallidum *(Dako^®^) and anti-herpes simplex virus type I and II antibodies, as well as anti-VCAM, anti-ICAM-1, anti-CD4 and anti-CD8 antibodies (all from Novocastra^®^) were used as primary antibodies.

The immunohistochemistry technique used included deparaffinization in warm xylol (37°C) and the use of methyl alcohol and H_2_O_2 _for the first endogenous peroxidase blocking, followed by distilled water and H_2_O_2 _for the second. Antigen retrieval was carried out using BioSB^® ^antigen unmasking solution with citrate in a water bath at 99°C for 20 minutes. The primary antibodies were incubated overnight at the recommended dilutions. EnVision^®^+Dual Link/Peroxidase, a dextran polymer from Dakocytomation^®^, was used for 30 minutes as the secondary antibody. Staining was developed by adding DAB chromogen and substrate to the slides, which were counterstained with Harris's hematoxylin. Positive and negative controls were used for all the reactions.

An Olympus BX50 microscope was used to identify positive immunohistochemical reactions. Slides were recorded as being positive or negative for the HIV p24 antigen, herpes simplex virus types I and II, *Treponema pallidum*, cytomegalovirus, *Toxoplasma gondii*, VCAM and ICAM-1. The readings for the slides with anti-CD4 and anti-CD8 were based on the number of cells per high-power field (HPF) that were positive for these markers. Thirty HPFs were counted and only positive cells that were inside the stroma villous were included.

For statistical analysis, cases were classified as either positive or negative for the p24 antigen (HIV), herpes simplex virus type I and II, *Treponema pallidum*, cytomegalovirus, *Toxoplasma gondii *and to VCAM and ICAM-1. To analyze the immunohistochemistry results for CD4 and CD8, we used the means and medians of the positive cell counts for each sample. The areas and perimeters of the placental villi from the HIV-seropositive and HIV-seronegative pregnant women were compared using the Student t-test for independent samples or the non-parametric Mann-Whitney test, as appropriate. The other variables were compared in a similar way using the Fisher exact test. Comparison, of the area and perimeter together was carried out using Rao's statistic. A significance level of p < 0.05 was used. The study was approved by the ethics committee of the Hospital de Clínicas, Curitiba, Brazil.

## Results

Approximately half (52%) of the study population was aged between 20 and 30 years. Pregnancies were carried to term by 77.19% and 63.33% of the HIV-seropositive and HIV-seronegative women, respectively (Table 1). Slightly over three-quarters (75.44%) of the newborns in the HIV-seropositive group weighed more than 2500 grams, while the corresponding figure for the HIV-seronegative group was 71.67% (Table 1). Analysis of gestational age and weight of newborns showed that there were no significant differences between the HIV-seropositive and HIV-seronegative groups (Table 2).

**Table 1:** Gestational age (weeks) and weight of the newborns (grams) data

GESTATIONAL AGE (WEEKS)	NUMBER OF CASES OF HIV-SEROPOSITIVE (n = 57)	NUMBER OF CASES OF HIV-SERONEGATIVE (n = 60)
25/30 WEEKS	3	9

31/36 WEEKS	10	13

37/42 WEEKS	44	38

WEIGHT OF THE NEWBORNS	NUMBER OF CASES OF HIV-SEROPOSITIVE	NUMBER OF CASES OF HIV-SERONEGATIVE

< 1000 GRAMS	2	6

BETWEEN 1000 AND 2500 GRAMS	12	11

› 2500 GRAMS	43	43

**Table 2:** Demographic profile of the pregnant women and morphometric data for the placental villi (in microns).

	MEAN OF HIV- SEROPOSITIVE GROUP	MEAN OF HIV- SERONEGATIVE GROUP	*p *VALUE
AGE OF THE PREGNANT WOMEN (YEARS)	26.68	26.11	0.694

GESTATIONAL AGE (WEEKS)	36.95	36.38	0.714

WEIGHT OF THE NEWBORNS (GRAMS)	2830.63	3071.52	0.166

AREA OF PLACENTAL VILLI (MICRONS)	3932.94	4339.93	0.014

PERIMETER OF PLACENTAL VILLI (MICRONS)	246.15	258.05	0.015

*KEY: *p = confidence interval <0.05.

Two of the newborns had HIV, and one of these died (the vertical transmission rate in this study was 3.51%).

Morphometric analysis showed that there were significant differences between the areas and perimeters of the villi in placentas from HIV-seropositive women and the corresponding areas and perimeters for HIV-seronegative women, the mean values for the latter group being higher (Table 2). These results suggest that the villi in placentas from the HIV-seronegative group may be larger than those from the HIV-seropositive group. Statistical analysis excluding cases in which pregnancies were not carried to term (gestational age of 25/36 weeks) failed to reveal any changes in the statistically significant data.

No statistically significant differences were found between the two groups of women in terms of the results for the immunohistochemical tests to detect p24 and antigens from *Toxoplasma gondii*, cytomegalovirus, herpes simplex I and II and *Treponema pallidum *(Table 3). Viral load measurements in the newborns whose placentas were positive for p24 (two placentas) failed to confirm maternal-fetal transmission. In contrast, the placentas of the two cases with fetal transmission were not positive for the p24 antigen.

**Table 3:** Results of immunohistochemical analysis for infections, adhesion molecules and subpopulations of T lymphocytes.

	HIV- SEROPOSITIVE GROUP	HIV- SEROPOSITIVE GROUP	*p *VALUE
p24	02	00	-

TOXOPLASMOSIS	00	00	-

SYPHILIS	01	00	-

CMV	00	00	-

HERPES	02	03	-

ICAM-1 POSITIVE	29	12	0.0013

ICAM-1 NEGATIVE	28	48	0.0013

VCAM POSITIVE	02	03	0.8300

VCAM NEGATIVE	55	57	0.8300

CD4 MEAN	1.47	1.49	0.6389

CD4 MEDIAN	0.67	0.75	0.6389

CD4 STANDARD DEVIATION	2.81	2.14	0.6389

CD8 MEAN	1.25	1.75	0.0316

CD8 MEDIAN	1.46	1.87	0.0316

CD8 STANDARD DEVIATION	1.13	1.11	0.0316

TOTAL NUMBER OF CASES	57	60	-

*KEY: *p24 = number of cases positive for p24; Toxoplasmosis, syphilis, herpes and CMV = number of cases positive for anti-*Toxoplasma gondii*, anti-cytomegalovirus, anti-CMV and anti-herpes antibodies; ICAM-1positive/negative = number of cases positive/negative for anti-ICAM-1; VCAM positive/negative = number of cases positive/negative for anti-VCAM; CD4/CD8 mean/median/standard deviation = mean and median CD4+ or CD8+ counts (and standard deviations) in placental villi, *p *= confidence interval of less than 0.05

ICAM-I was expressed both in vessels of the villous stroma and in Hofbauer cells. Expression of this molecule was observed in a larger percentage of cases in the HIV-seropositive group (57%), compared with only 21% of the cases in the control group. VCAM-1 was expressed in a small number of cases in both groups (3.6% in the seropositive group and 5.2% in the seronegative group) and was always observed in the vessels of the villous stroma (table 3).

CD4+ and CD8+ cell counts showed that there was a greater prevalence of CD8+ T cells in the HIV-seropositive women. No statistically significant difference in the number of CD4+ cells was observed in the placentas analyzed. CD4+ T cells were inconspicuous in all the cases, and the cells with the greatest positivity for CD4 were Hofbauer cells. The median CD8+ T cell count in HIV-seropositive pregnant women was 1.87 per HPF, and the corresponding figure in the control group was 1.46. This difference was statistically significant (p = 0.03) (Table 3).

Analysis of the viral loads revealed that these were measured between three and six times for most of the patients and that 54.1% had an initial viral load, during the first trimester of gestation, of less than 1000 copies. Taking the average of the three main viral loads measured during the pregnancies, we found that 72% of the women had a mean value of more than 2000 copies (Table 4). Comparison of the area and perimeter of the villi, ICAM-1 expression and CD8+ T-cell concentration with mean patient viral load failed to reveal any statistically significant correlations.

**Table 4:** Viral load data for seropositive patients analyzed in this study

NUMBER OF VIRAL COPIES	INITIAL VIRAL LOAD		4^TH ^VIRAL LOAD		MEAN OF 3 VIRAL LOADS		MEAN OF ALL THE VIRAL LOADS	
	**NUMBER**	**PERCENTAGE**	**NUMBER**	**PERCENTAGE**	**NUMBER**	**PERCENTAGE**	**NUMBER**	**PERCENTAGE**

<1000	13	54.1	9	22.5	5	13.5	5	13.8

<2000	1	4.1	3	7.5	5	13.5	1	2.7

<10000	5	20.8	11	27.5	13	35.1	10	27.7

>10000	5	20.8	17	42.5	14	37.8	20	55.5

TOTAL	24		40		37		36	

KEY: Number/Percentage = number and percentage of pregnant women for whom the measurements of the first and fourth viral loads, the mean of the first three loads and the mean of all the loads were equal to the viral load shown.

The use of appropriate antiretroviral therapy in the HIV-seropositive women and its relationship with markers of the disease were also analyzed. Antiretroviral therapy administered for at least one month no later than one month before the birth was considered appropriate therapy (therapy +), while antiretroviral therapy administered for less than one month prior to the birth or failure to administer therapy was considered inappropriate or absent therapy (therapy -). No statistically significant correlation was observed between CD8+ T-cell count or the area and perimeter of the villi and the use of antiretroviral drugs. However, a correlation was observed with ICAM-1 expression, which was higher in the group that had received therapy (p = 0.03), as well as with viral load, which was lower in the same group (p = 0.01) (Table 5).

**Table 5:** Correlation between the use of antiretroviral therapy and immunohistochemical, morphometric and viral load data.

	THERAPY +	THERAPY -	*p *VALUE
ICAM1+	15	12	0.0322

CD8 + MEDIAN	1.75	1.5	0.6324

AREA MEDIAN	3711.84	3972.13	0.2940

PERIMETER MEDIAN	243.21	248.66	0.5580

MEDIAN OF 3 VIRAL LOAD	5841.67	19446.67	0.0165

TOTAL	35	16	

KEY: therapy += patients who had antiretroviral therapy for at least 1 month before giving birth; therapy - = patients who only had loading doses or did not undergo antiretroviral therapy; ICAM-1+ = number of cases with immunohistochemical expression of ICAM-1; median CD8+ = median CD8+ T cell count; median area/perimeter = median of area and perimeter in microns of placental villi; median of 3 viral loads = median of the three first viral loads; *p *= confidence interval of less than 0.05.

The statistically significant results continued to be statistically signficant when cases that were positive for infectious agents as confirmed by immunohistochemical reaction were excluded from the statistical analysis.

## Discussion

The importance of pathological examination of the placenta in HIV-seropositive mothers has been recognized by health professionals for some years, as it allows several diseases to be diagnosed and also provides additional information and statistical data[5]. In general, the placentas of mothers who are only HIV-seropositive have a normal macroscopic and microscopic appearance, as was the case in the patients selected for this study.^5 ^However, as observed in our study, when samples from these normal placentas are analyzed using more specific and sensitive methods such as immunohistochemistry and molecular biology, they do not yield normal results[6].

The morphometric findings of this study also revealed changes in the diameter and perimeter of the placental villi of HIV-seropositive women that were suggestive of changes in villous maturation and may have been caused by the viral infection itself and/or even use of antiretroviral drugs.

The frequency of positive immunohistochemical results for microorganisms that cause intrauterine infections was the same in placentas from HIV-seropositive women and placentas from HIV-seronegative women, a finding that has been reported in the literature, and this fact could be particularly true in the normal placenta samples of HIV-seropositive pregnant[7].

Our results for immunohistochemical testing revealed the presence of HIV viral proteins (p24) in two samples even in the absence of histological lesions[6,8]. The low number of cases in which p24 was detected (2/57) may be related to the low expression of this marker, since it can cross-react with endogenous antigens, leading to false positive or false negative results[3,9]. However, the absence of positive results in the control group indicates that the antibody against p24 used in the test did not yield false positive results. Furthermore, studies have shown that the virus is not detected by the anti-p24 antibody in patients undergoing antiretroviral treatment[6]. Although Lee[10] and Tschening-Casper[11] detected HIV sequences by PCR in placental cells from HIV-seropositive women who were receiving antiretroviral therapy, the placentas did not have any macroscopic or histological abnormalities, and immunohistochemical analysis failed to detect HIV-1 p24 antigens.

In our study we observed positive reactions in some cytotrophoblast cells and Hofbauer cells in the placentas that were positive for p24. When human placentas infected with HIV in utero or in vitro were analyzed by PCR, the HIV-1 virus was found to be distributed mainly in syncytiotrophoblast and Hofbauer cells [3]. In addition, in several studies HIV-1 sequences were detected in both chorionic villi and trophoblasts in all the placentas analyzed, indicating that the virus sequences are always present in the placentas of these patients, even in the absence of morphological alterations and immunohistochemical expression characteristic of HIV[12,13].

The elevated expression of ICAM-1 in the placentas of HIV-seropositive women observed in this study may indicate that this molecule can act as an inflammatory marker of this disease[14]. Similarly, the elevated expression of this molecule in the vascular endothelium and the cytoplasm of Hofbauer cells in placental villi may indicate that the HIV virus has passed through the placental barrier, since ICAM is the molecule that mediates the entry of the virus into the macrophage. After the virus has penetrated the placental villi, it can adsorb to the surface of the ICAM molecules and mediate infections in T cells and Hofbauer cells[15]. The presence of the virus in the placental villi is also suggested the by large number of CD8+ T cells in the HIV-seropositive women (p = 0.03).

There was no statistically significant difference in the expression of CD4+ cells between the two groups. CD4+ T cells were inconspicuous inside the chorionic villi. However, large numbers of Hofbauer cells were positive for CD4. CD4+ T cells inside the chorionic villi may mediate control of HIV infection and possible another infections in the placenta[16].

The binding of HIV viral particles to CD4+ receptors on the surface of Hofbauer cells after the particles have adsorbed to ICAM molecules may be the main transmission path for HIV inside the villous stroma in placentas. Once adsorbed to ICAM molecules, the viral particles can bind to Hofbauer cells by means of these molecules and use their migratory ability to reach the fetal vessels and then infect the conceptus's cells. ^15 ^In light of our results for CD4+ count and ICAM-1 expression in placental villi, it is reasonable to suppose that entry of the virus into the fetal circulatory system may be intimately related to the CD4+ Hofbauer cells found in the villous stroma[17].

CD8+ T cells were present in greater quantities in the placentas of the HIV-seropositive women, probably because they are associated with anti-viral immune responses against HIV. Although the early villitis of unknown etiology are defined by villi injury, presence of maternal CD8+ T cells and presence of hyperplasic Hofbauer cells, there were no morphological changes compatible with villitis in this study[18]. It might represent a very early stage of this entity which only would show immunohistochemical changes. Otherwise, these immunohistochemical findings might represent early changes of a specific villitis by HIV, but not a villitis of unknown etiology.

The increased immunohistochemical expression of CD8 in the placentas of HIV-seropositive patients may be intimately related to the presence of cytotoxic CD8+ T cells specific to HIV. Acute HIV infection is characterized by an increased number of cytotoxic CD8+ T cells involved in the control of the viremia. These lymphocytes play an important role in acute and chronic HIV infection, and an increase in their number may contribute significantly to protection against intrauterine transmission of HIV[19]. Cells that contain HIV gp120 glycoprotein (an envelope antigen) in their plasma membrane are recognized by activated cytotoxic CD8+ T lymphocytes, which destroy not only the virus but also the whole cell by releasing cytolytic substances [17]. As well as mediating cytolytic activity, CD8+ T cells can suppress HIV by secreting a factor, or set of factors, known as CD8 cell antiviral factor (CAF). CAF can block CCR5 and CXCR4 receptors by transcription regulation and consequently prevent viral replication [19]. These CD8+ T cells are able to produce a cytokine response similar to that in adults. In congenital infection, newborns can develop a mature CD8+ T-cell response to cytomegalovirus similar to that detected in adults [20]. Another factor that may be associated with the presence of CD8+ T cells in the placentas of HIV-seropositive women is that these cells are able to produce antiviral factors mediated by HIV-specific IgG stimulation. These specific antibodies are transferred efficiently from the mother to the conceptus through the placenta[16,21].

The low expression of VCAM in both groups may be associated with the low stimulation of cytokines that mediate the expression of VCAM on the cell surface. If it is assumed that these placentas did not have active infections, then they did not need VCAM to be activated to recruit activated T cells[14].

Analysis of the viral load counts showed that 72% of the pregnant women had mean viral loads in excess of two thousand copies. This may indicate that the study population had not received effective treatment as in the majority of cases the viral load was higher than 50 copies (Table 4). Our results also showed that the viral loads did not affect expression of ICAM-1, CD8+ T-cell concentrations or the area or perimeter of the placental villi.

When we analyzed the medical records, we separated patients into those who had received treatment for an appropriate period and those who had not. When these two groups were analyzed, no correlation was found with CD8+ cells counts and the area or perimeter of the placental villi. In other words, antiretroviral therapy did not appear to have been responsible for the increased CD8+ cells count or the reduced perimeter and area of placental villi in the HIV-seropositive women. However, a correlation was identified between antiretroviral therapy and immunohistochemical expression of ICAM-1, which appeared to be higher in patients who had received this therapy. There was also a correlation between antiretroviral therapy and mean viral load, which, as expected, was smaller in this group although it was not less than 50 copies, the limit of detection defined by WHO.

## Conclusions

In conclusion, the morphometric data show that seropositive placentas tend to have smaller villi than seronegative ones. In addition, immunohistochemical examination for infectious agents helped to identify cases that were positive for microorganisms (6/112) that routine pathological tests had failed to detect. The anti-p24 antibody proved not to have a high rate of detection of HIV viral protein in this study (2/57). Correlation of immunohistochemical expression of CD8+ T cells and ICAM-1 with the presence of HIV in the placenta revealed that these markers might be acting as biomarkers of nonspecific inflammation. Otherwise, they might be acting as biomarkers for specific injured by HIV infection or another unknown coinfeccion.

## Competing interests

The authors declare that they have no competing interests.

## Authors' contributions

VMN, intellectual author, wrote the paper. EB: intellectual author, wrote the paper. APC: immunohistochemistry tests. CRP: immunohistochemistry tests. MM: immunohistochemistry tests. MGS: morphometric results. CDAS: morphometric results. KA: morphometric results. LLGS: immunohistochemistry results. AOS: immunohistochemistry results. CRC: newborns data. LN: intellectual author, wrote the paper. All authors read and approved the final manuscript.
